# Molecular Study of Sudden Cardiac Death

**DOI:** 10.3390/ijms25126366

**Published:** 2024-06-08

**Authors:** Sorin Hostiuc

**Affiliations:** Department of Legal Medicine and Bioethics, Faculty of Dentistry, Carol Davila University of Medicine and Pharmacy, 042122 Bucharest, Romania; soraer@gmail.com

## 1. Editorial

The aim of the Special Issue “Molecular study of sudden cardiac death” was to gather new studies on the molecular biology of cardiac death, from both a fundamental and clinical perspective.

Causes of a cardiac nature remain among the most prevalent causes of death globally [1] despite ever-increasing financing strategies aimed at decreasing their incidence [1,2]. Even though the most important risk factors for cardiovascular disorders encompass metabolic, environmental, behavioral, and social factors [3], there is increasing evidence to support the hypothesis that genetic factors play important roles, both directly and indirectly, by modulating other types of factors [4,5]. The articles published as part of this Special Issue were mainly directed toward the analysis of the genetic and molecular profiling of cardiovascular disorders.

One of the most important methods employed in the past few years to establish large-scale correlations in the study of various cardiovascular disorders is big data analysis [6,7,8], which has been, as of late, complemented by artificial intelligence tools [9,10]. Cardiovascular disorders are one of the areas in which big data analysis has been extensively used in the last few years, through the employment of predictive frameworks [11,12,13], to detect correlations with other, non-cardiovascular pathologies [14], predict the risk of cardiovascular disorders, etc. The authors of one such study attempted to evaluate the genes associated with the risk of myocardial infarction, with 28 genes that increase overall risk being found, including PAI-1, CX-37, IL-18, LTA, LGALS2, LDLR, and APOA5 [15]. By identifying genes associated with increased risk, using big data analysis, more targeted studies may be performed to evaluate the actual connection in depth and ascertain whether it is causal or only associative.

A major cause of cardiac death is represented by cardiac arrhythmias, which often have, at least partially, a genetic etiology [16,17,18]. There have been numerous articles published over the past few decades focusing specifically on the genetic component of cardiac arrhythmias, with the results of such studies showing a high number of potential causal associations. One such study, published as part of the present Special Issue, performed by Hartman et al. aimed to investigate the electrophysiological contribution of Na_V_1.8 (a voltage-gated sodium channel) to the generation of atrial arrhythmogenesis by using a human SCN10A knock-out stem cell model. The results of the study conclusively showed that Na_V_1.8 inhibition can modulate pro-arrhythmogenic triggers in atrial cardiomyocytes, suggesting its potential usefulness as a target in arrhythmogenic strategies [19]. The results of this particular study thus confirm the results of other studies in this research field, which may soon have significant therapeutic implications [20].

Within the same area of study, Mircea et al. performed a comprehensive review evaluating some promising therapies for atrial fibrillation and ventricular tachycardia [21], with the authors focusing their attention on novel therapeutic agents, such as vernakalant, which has been shown to improve the outcomes of patients suffering from new-onset atrial fibrillation [22], or azimilide, which blocks both rapid and slowed delayed inward rectifier channels [23], with it being shown to decrease the reoccurrence risk of ventricular fibrillation [24].

Some sudden cardiac deaths with no known cause (the so-called “negative autopsies” in cases of sudden death) have been shown to have a genetic cause. However, the number of exonic pathogenic variants or likely pathogenic variants account for less than one-third of all negative autopsies [25], making the study of variants of unknown origin a very important and hot topic of research in this area. Coll et al. performed a study in an attempt to identify unpredicted aberrant splicing products in samples from subjects who died suddenly from cardiac causes. The study authors focused on the issue of potential splice site variants, which are extremely difficult to interpret and are often overlooked during genetic tests for cardiovascular disorders [26].

## 2. Conclusions

As can be seen from the results of the aforementioned studies, there are still many unknowns surrounding the genetic etiology of sudden cardiac death in particular and cardiovascular disorders in general. A proper understanding of the intimate, underlying mechanisms involved may aid in decreasing mortality, decreasing the overall burden on the healthcare system, and, ultimately, allowing all of us to live better and longer lives.
